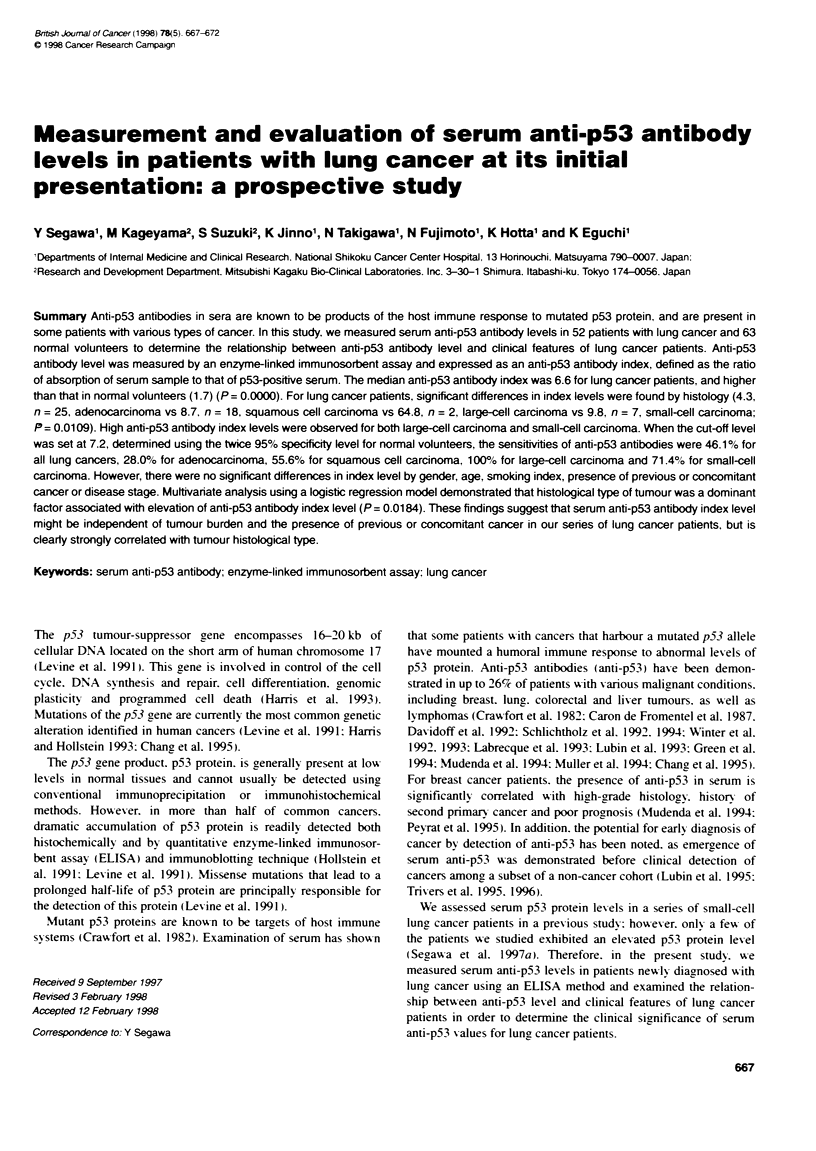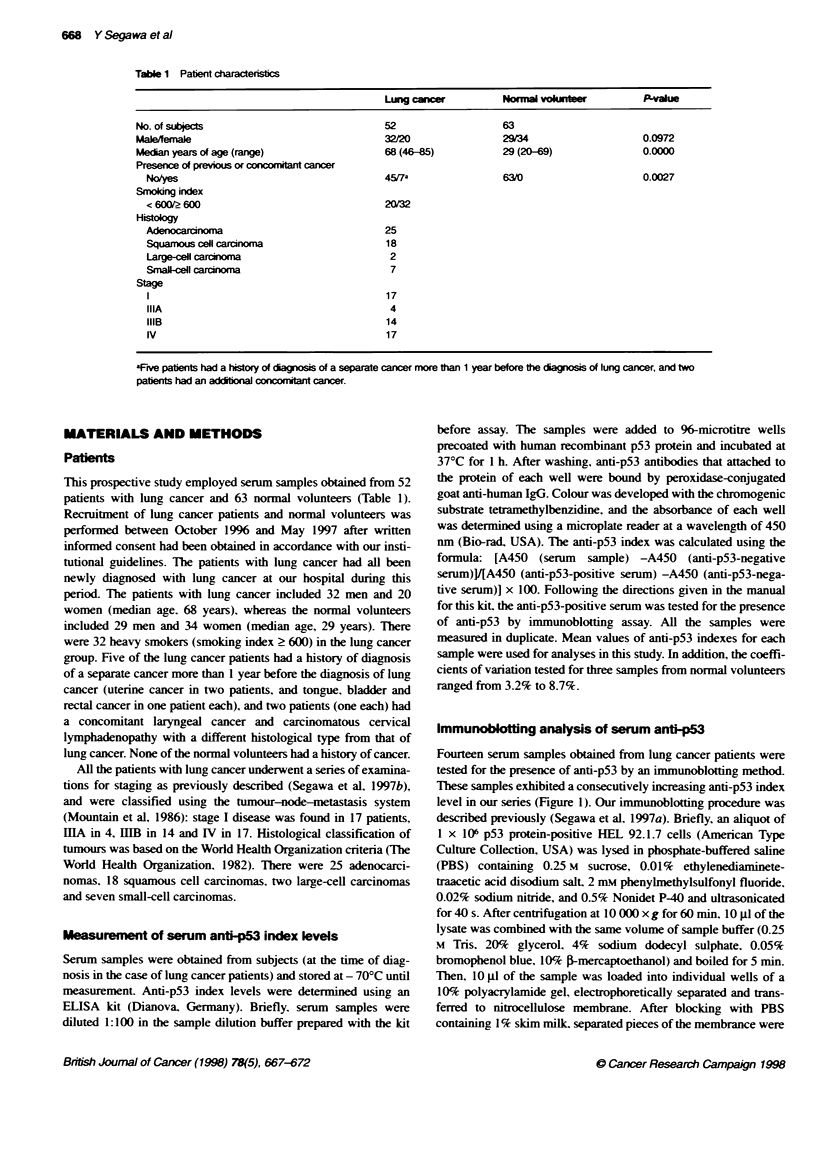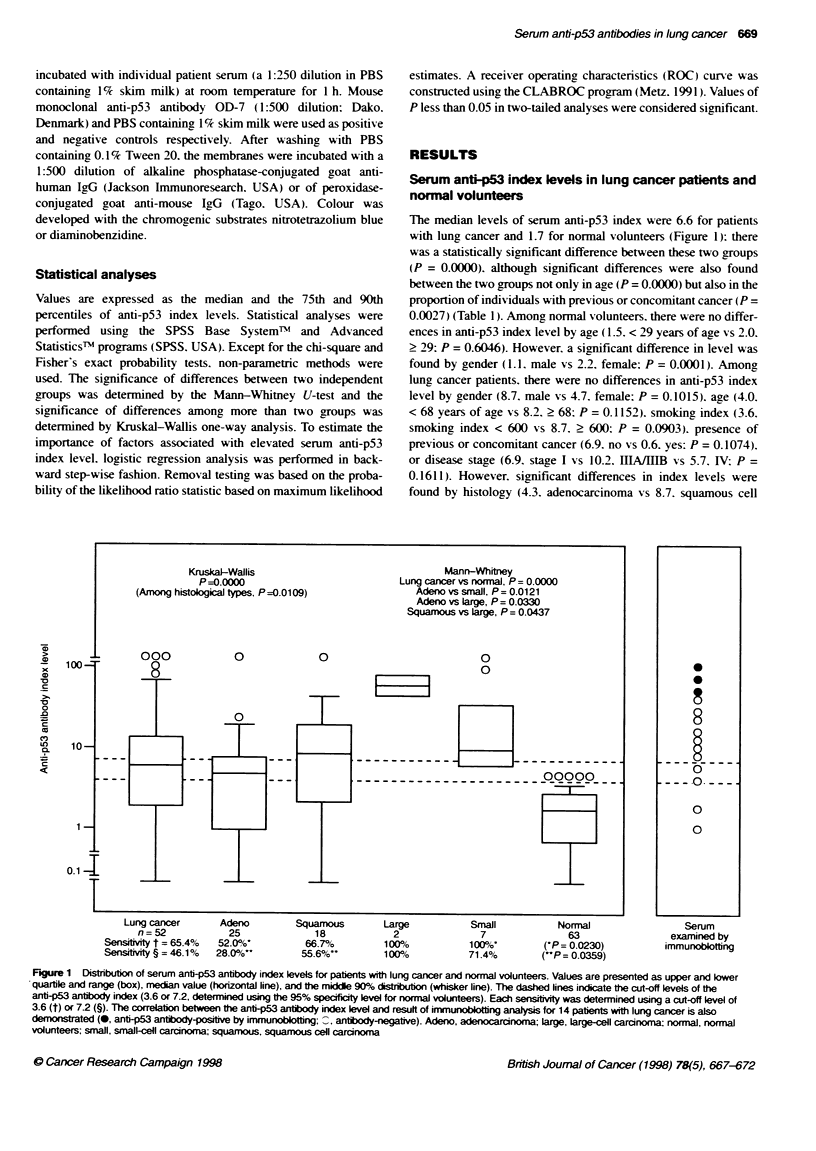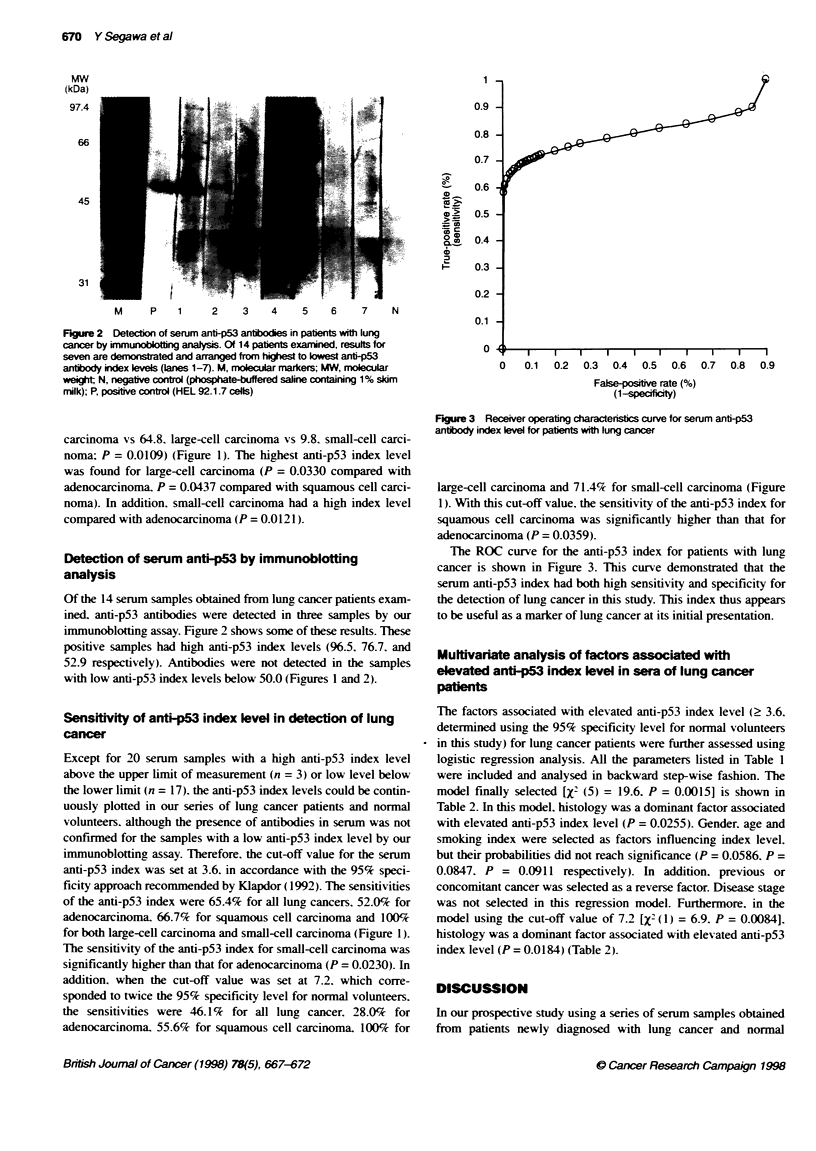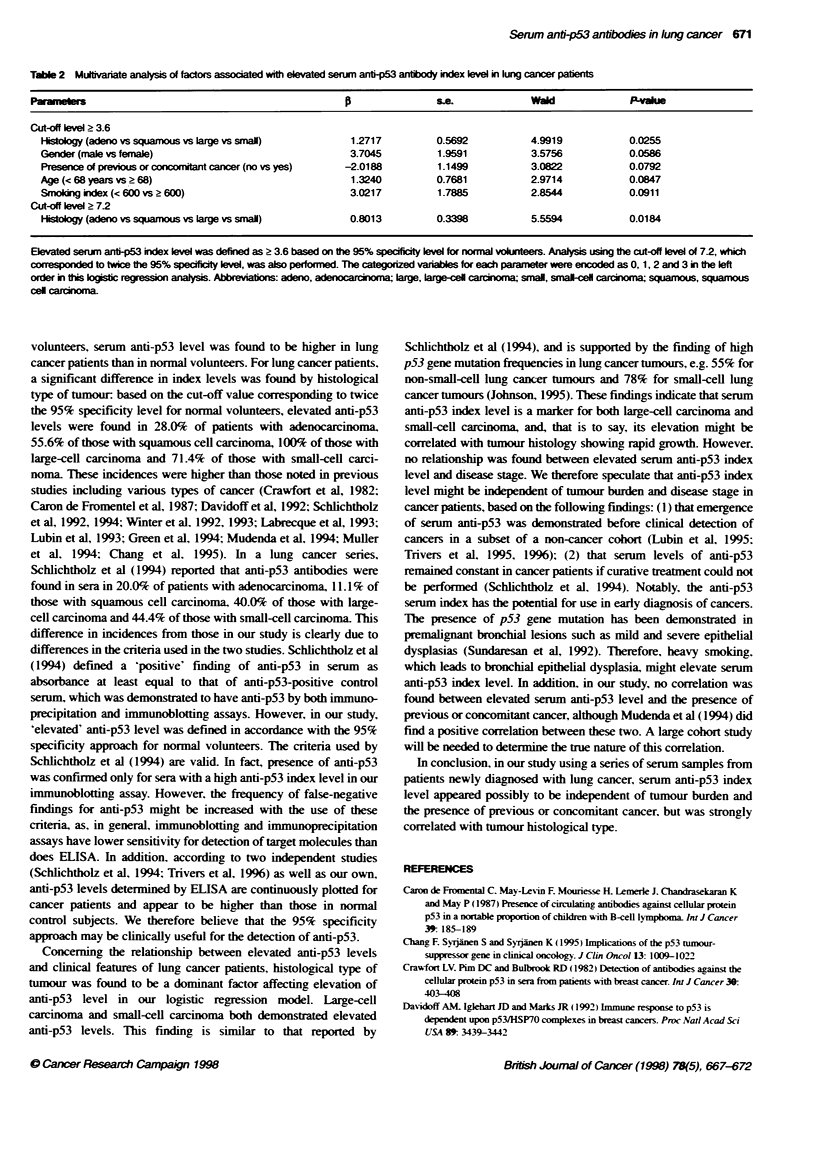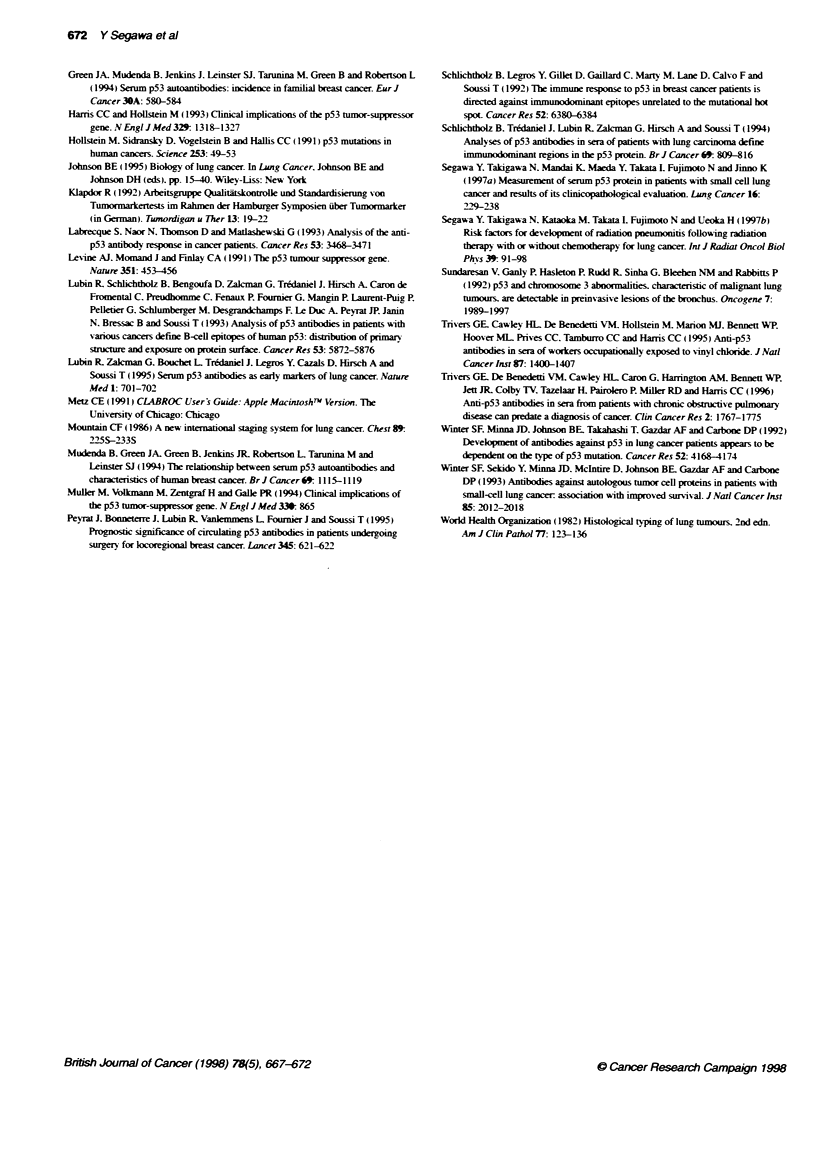# Measurement and evaluation of serum anti-p53 antibody levels in patients with lung cancer at its initial presentation: a prospective study.

**DOI:** 10.1038/bjc.1998.557

**Published:** 1998-09

**Authors:** Y. Segawa, M. Kageyama, S. Suzuki, K. Jinno, N. Takigawa, N. Fujimoto, K. Hotta, K. Eguchi

**Affiliations:** Department of Internal Medicine, National Shikoku Cancer Center Hospital, Matsuyama, Japan.

## Abstract

**Images:**


British Journal of Cancer (1998) 78(5). 667–672
© 1998 Cancer Research Campaign

### Measurement and evaluation of serum anti-p53 antibody levels in patients with lung cancer at its initial presentation: a prospective study

**Y Segawa[1], M Kageyama[2], S Suzuki[2], K Jinno[1], N Takigawa[1], N Fujimoto[1], K Hotta[1] and K Eguchi[1]**

[1]Departments of Internal Medicine and Clinical Research. National Shikoku Cancer Center Hospital. 13 Horinouchi. Matsuyama 790–0007. Japan: [2]Research and Development Department. Mitsubishi Kagaku Bio-Clinical Laboratories. Inc. 3-30-1 Shimura. Itabashi-ku. Tokyo 174–0056. Japan

**Summary** Anti-p53 antibodies in sera are known to be products of the host immune response to mutated p53 protein, and are present in some patients with various types of cancer. In this study, we measured serum anti-p53 antibody levels in 52 patients with lung cancer and 63 normal volunteers to determine the relationship between anti-p53 antibody level and clinical features of lung cancer patients. Anti-p53 antibody level was measured by an enzyme-linked immunosorbent assay and expressed as an anti-p53 antibody index, defined as the ratio of absorption of serum sample to that of p53-positive serum. The median anti-p53 antibody index was 6.6 for lung cancer patients, and higher than that in normal volunteers (1.7) ($P$ = 0.0000). For lung cancer patients, significant differences in index levels were found by histology (4.3, $n$ = 25, adenocarcinoma vs 8.7, $n$ = 18, squamous cell carcinoma vs 64.8, $n$ = 2, large-cell carcinoma vs 9.8, $n$ = 7, small-cell carcinoma; $P$ = 0.0109). High anti-p53 antibody index levels were observed for both large-cell carcinoma and small-cell carcinoma. When the cut-off level was set at 7.2, determined using the twice 95% specificity level for normal volunteers, the sensitivities of anti-p53 antibodies were 46.1% for all lung cancers, 28.0% for adenocarcinoma, 55.6% for squamous cell carcinoma, 100% for large-cell carcinoma and 71.4% for small-cell carcinoma. However, there were no significant differences in index level by gender, age, smoking index, presence of previous or concomitant cancer or disease stage. Multivariate analysis using a logistic regression model demonstrated that histological type of tumour was a dominant factor associated with elevation of anti-p53 antibody index level ($P$ = 0.0184). These findings suggest that serum anti-p53 antibody index level might be independent of tumour burden and the presence of previous or concomitant cancer in our series of lung cancer patients, but is clearly strongly correlated with tumour histological type.

**Keywords:** serum anti-p53 antibody; enzyme-linked immunosorbent assay; lung cancer

The *p53* tumour-suppressor gene encompasses 16–20 kb of cellular DNA located on the short arm of human chromosome 17 (Levine et al. 1991). This gene is involved in control of the cell cycle. DNA synthesis and repair. cell differentiation. genomic plasticity and programmed cell death (Harris et al. 1993). Mutations of the *p53* gene are currently the most common genetic alteration identified in human cancers (Levine et al. 1991: Harris and Hollstein 1993: Chang et al. 1995).

The *p53* gene product. p53 protein. is generally present at low levels in normal tissues and cannot usually be detected using conventional immunoprecipitation or immunohistochemical methods. However. in more than half of common cancers. dramatic accumulation of p53 protein is readily detected both histochemically and by quantitative enzyme-linked immunosorbent assay (ELISA) and immunoblotting technique (Hollstein et al. 1991: Levine et al. 1991). Missense mutations that lead to a prolonged half-life of p53 protein are principally responsible for the detection of this protein (Levine et al. 1991).

Mutant p53 proteins are known to be targets of host immune systems (Crawfort et al. 1982). Examination of serum has shown that some patients with cancers that harbour a mutated *p53* allele have mounted a humoral immune response to abnormal levels of p53 protein. Anti-p53 antibodies (anti-p53) have been demonstrated in up to 26% of patients with various malignant conditions. including breast. lung. colorectal and liver tumours. as well as lymphomas (Crawfort et al. 1982: Caron de Fromentel et al. 1987. Davidoff et al. 1992: Schlichtholz et al. 1992. 1994: Winter et al. 1992. 1993: Labrecque et al. 1993: Lubin et al. 1993: Green et al. 1994: Mudenda et al. 1994: Muller et al. 1994: Chang et al. 1995). For breast cancer patients. the presence of anti-p53 in serum is significantly correlated with high-grade histology. history of second primary cancer and poor prognosis (Mudenda et al. 1994: Peyrat et al. 1995). In addition. the potential for early diagnosis of cancer by detection of anti-p53 has been noted. as emergence of serum anti-p53 was demonstrated before clinical detection of cancers among a subset of a non-cancer cohort (Lubin et al. 1995: Trivers et al. 1995. 1996).

We assessed serum p53 protein levels in a series of small-cell lung cancer patients in a previous study: however. only a few of the patients we studied exhibited an elevated p53 protein level (Segawa et al. 1997*a*). Therefore. in the present study. we measured serum anti-p53 levels in patients newly diagnosed with lung cancer using an ELISA method and examined the relationship between anti-p53 level and clinical features of lung cancer patients in order to determine the clinical significance of serum anti-p53 values for lung cancer patients.

*Received 9 September 1997*
*Revised 3 February 1998*
*Accepted 12 February 1998*

*Correspondence to:* Y Segawa

**Table 1** Patient characteristics

| | Lung cancer | Normal volunteer | P-value |
|---|---|---|---|
| No. of subjects | 52 | 63 | |
| Male/female | 32/20 | 29/34 | 0.0972 |
| Median years of age (range) | 68 (46–85) | 29 (20–69) | 0.0000 |
| Presence of previous or concomitant cancer | | | |
| No/yes | 45/7[a] | 63/0 | 0.0027 |
| Smoking index | | | |
| < 600/≥ 600 | 20/32 | | |
| Histology | | | |
| Adenocarcinoma | 25 | | |
| Squamous cell carcinoma | 18 | | |
| Large-cell carcinoma | 2 | | |
| Small-cell carcinoma | 7 | | |
| Stage | | | |
| I | 17 | | |
| IIIA | 4 | | |
| IIIB | 14 | | |
| IV | 17 | | |

[a]Five patients had a history of diagnosis of a separate cancer more than 1 year before the diagnosis of lung cancer, and two patients had an additional concomitant cancer.

### MATERIALS AND METHODS

### Patients

This prospective study employed serum samples obtained from 52 patients with lung cancer and 63 normal volunteers (Table 1). Recruitment of lung cancer patients and normal volunteers was performed between October 1996 and May 1997 after written informed consent had been obtained in accordance with our institutional guidelines. The patients with lung cancer had all been newly diagnosed with lung cancer at our hospital during this period. The patients with lung cancer included 32 men and 20 women (median age, 68 years), whereas the normal volunteers included 29 men and 34 women (median age, 29 years). There were 32 heavy smokers (smoking index ≥ 600) in the lung cancer group. Five of the lung cancer patients had a history of diagnosis of a separate cancer more than 1 year before the diagnosis of lung cancer (uterine cancer in two patients, and tongue, bladder and rectal cancer in one patient each), and two patients (one each) had a concomitant laryngeal cancer and carcinomatous cervical lymphadenopathy with a different histological type from that of lung cancer. None of the normal volunteers had a history of cancer.

All the patients with lung cancer underwent a series of examinations for staging as previously described (Segawa et al, 1997*b*), and were classified using the tumour–node–metastasis system (Mountain et al, 1986): stage I disease was found in 17 patients, IIIA in 4, IIIB in 14 and IV in 17. Histological classification of tumours was based on the World Health Organization criteria (The World Health Organization, 1982). There were 25 adenocarcinomas, 18 squamous cell carcinomas, two large-cell carcinomas and seven small-cell carcinomas.

### Measurement of serum anti-p53 index levels

Serum samples were obtained from subjects (at the time of diagnosis in the case of lung cancer patients) and stored at – 70°C until measurement. Anti-p53 index levels were determined using an ELISA kit (Dianova, Germany). Briefly, serum samples were diluted 1:100 in the sample dilution buffer prepared with the kit before assay. The samples were added to 96-microtitre wells precoated with human recombinant p53 protein and incubated at 37°C for 1 h. After washing, anti-p53 antibodies that attached to the protein of each well were bound by peroxidase-conjugated goat anti-human IgG. Colour was developed with the chromogenic substrate tetramethylbenzidine, and the absorbance of each well was determined using a microplate reader at a wavelength of 450 nm (Bio-rad, USA). The anti-p53 index was calculated using the formula: [A450 (serum sample) –A450 (anti-p53-negative serum)]/[A450 (anti-p53-positive serum) –A450 (anti-p53-negative serum)] × 100. Following the directions given in the manual for this kit, the anti-p53-positive serum was tested for the presence of anti-p53 by immunoblotting assay. All the samples were measured in duplicate. Mean values of anti-p53 indexes for each sample were used for analyses in this study. In addition, the coefficients of variation tested for three samples from normal volunteers ranged from 3.2% to 8.7%.

### Immunoblotting analysis of serum anti-p53

Fourteen serum samples obtained from lung cancer patients were tested for the presence of anti-p53 by an immunoblotting method. These samples exhibited a consecutively increasing anti-p53 index level in our series (Figure 1). Our immunoblotting procedure was described previously (Segawa et al, 1997*a*). Briefly, an aliquot of 1 × $10^6$ p53 protein-positive HEL 92.1.7 cells (American Type Culture Collection, USA) was lysed in phosphate-buffered saline (PBS) containing 0.25 M sucrose, 0.01% ethylenediaminetetraacetic acid disodium salt, 2 mM phenylmethylsulfonyl fluoride, 0.02% sodium nitride, and 0.5% Nonidet P-40 and ultrasonicated for 40 s. After centrifugation at 10 000 × *g* for 60 min, 10 μl of the lysate was combined with the same volume of sample buffer (0.25 M Tris, 20% glycerol, 4% sodium dodecyl sulphate, 0.05% bromophenol blue, 10% β-mercaptoethanol) and boiled for 5 min. Then, 10 μl of the sample was loaded into individual wells of a 10% polyacrylamide gel, electrophoretically separated and transferred to nitrocellulose membrane. After blocking with PBS containing 1% skim milk, separated pieces of the membrance were

© Cancer Research Campaign 1998

incubated with individual patient serum (a 1:250 dilution in PBS containing 1% skim milk) at room temperature for 1 h. Mouse monoclonal anti-p53 antibody OD-7 (1:500 dilution: Dako, Denmark) and PBS containing 1% skim milk were used as positive and negative controls respectively. After washing with PBS containing 0.1% Tween 20, the membranes were incubated with a 1:500 dilution of alkaline phosphatase-conjugated goat anti-human IgG (Jackson Immunoresearch, USA) or of peroxidase-conjugated goat anti-mouse IgG (Tago, USA). Colour was developed with the chromogenic substrates nitrotetrazolium blue or diaminobenzidine.

### Statistical analyses

Values are expressed as the median and the 75th and 90th percentiles of anti-p53 index levels. Statistical analyses were performed using the SPSS Base System™ and Advanced Statistics™ programs (SPSS, USA). Except for the chi-square and Fisher's exact probability tests, non-parametric methods were used. The significance of differences between two independent groups was determined by the Mann–Whitney *U*-test and the significance of differences among more than two groups was determined by Kruskal–Wallis one-way analysis. To estimate the importance of factors associated with elevated serum anti-p53 index level, logistic regression analysis was performed in backward step-wise fashion. Removal testing was based on the probability of the likelihood ratio statistic based on maximum likelihood estimates. A receiver operating characteristics (ROC) curve was constructed using the CLABROC program (Metz, 1991). Values of *P* less than 0.05 in two-tailed analyses were considered significant.

### RESULTS

### Serum anti-p53 index levels in lung cancer patients and normal volunteers

The median levels of serum anti-p53 index were 6.6 for patients with lung cancer and 1.7 for normal volunteers (Figure 1): there was a statistically significant difference between these two groups ($P$ = 0.0000), although significant differences were also found between the two groups not only in age ($P$ = 0.0000) but also in the proportion of individuals with previous or concomitant cancer ($P$ = 0.0027) (Table 1). Among normal volunteers, there were no differences in anti-p53 index level by age (1.5, < 29 years of age vs 2.0, ≥ 29: $P$ = 0.6046). However, a significant difference in level was found by gender (1.1, male vs 2.2, female: $P$ = 0.0001). Among lung cancer patients, there were no differences in anti-p53 index level by gender (8.7, male vs 4.7, female: $P$ = 0.1015), age (4.0, < 68 years of age vs 8.2, ≥ 68: $P$ = 0.1152), smoking index (3.6, smoking index < 600 vs 8.7, ≥ 600: $P$ = 0.0903), presence of previous or concomitant cancer (6.9, no vs 0.6, yes: $P$ = 0.1074), or disease stage (6.9, stage I vs 10.2, IIIA/IIIB vs 5.7, IV: $P$ = 0.1611). However, significant differences in index levels were found by histology (4.3, adenocarcinoma vs 8.7, squamous cell

**Figure 1** Distribution of serum anti-p53 antibody index levels for patients with lung cancer and normal volunteers. Values are presented as upper and lower quartile and range (box), median value (horizontal line), and the middle 90% distribution (whisker line). The dashed lines indicate the cut-off levels of the anti-p53 antibody index (3.6 or 7.2, determined using the 95% specificity level for normal volunteers). Each sensitivity was determined using a cut-off level of 3.6 (†) or 7.2 (§). The correlation between the anti-p53 antibody index level and result of immunoblotting analysis for 14 patients with lung cancer is also demonstrated (●, anti-p53 antibody-positive by immunoblotting; ○, antibody-negative). Adeno, adenocarcinoma; large, large-cell carcinoma; normal, normal volunteers; small, small-cell carcinoma; squamous, squamous cell carcinoma

© Cancer Research Campaign 1998

**Figure 2** Detection of serum anti-p53 antibodies in patients with lung cancer by immunoblotting analysis. Of 14 patients examined, results for seven are demonstrated and arranged from highest to lowest anti-p53 antibody index levels (lanes 1–7). M, molecular markers; MW, molecular weight; N, negative control (phosphate-buffered saline containing 1% skim milk); P, positive control (HEL 92.1.7 cells)

**Figure 3** Receiver operating characteristics curve for serum anti-p53 antibody index level for patients with lung cancer carcinoma vs 64.8, large-cell carcinoma vs 9.8, small-cell carcinoma; $P = 0.0109$) (Figure 1). The highest anti-p53 index level was found for large-cell carcinoma ($P = 0.0330$ compared with adenocarcinoma, $P = 0.0437$ compared with squamous cell carcinoma). In addition, small-cell carcinoma had a high index level compared with adenocarcinoma ($P = 0.0121$).

### Detection of serum anti-p53 by immunoblotting analysis

Of the 14 serum samples obtained from lung cancer patients examined, anti-p53 antibodies were detected in three samples by our immunoblotting assay. Figure 2 shows some of these results. These positive samples had high anti-p53 index levels (96.5, 76.7, and 52.9 respectively). Antibodies were not detected in the samples with low anti-p53 index levels below 50.0 (Figures 1 and 2).

### Sensitivity of anti-p53 index level in detection of lung cancer

Except for 20 serum samples with a high anti-p53 index level above the upper limit of measurement ($n = 3$) or low level below the lower limit ($n = 17$), the anti-p53 index levels could be continuously plotted in our series of lung cancer patients and normal volunteers, although the presence of antibodies in serum was not confirmed for the samples with a low anti-p53 index level by our immunoblotting assay. Therefore, the cut-off value for the serum anti-p53 index was set at 3.6, in accordance with the 95% specificity approach recommended by Klapdor (1992). The sensitivities of the anti-p53 index were 65.4% for all lung cancers, 52.0% for adenocarcinoma, 66.7% for squamous cell carcinoma and 100% for both large-cell carcinoma and small-cell carcinoma (Figure 1). The sensitivity of the anti-p53 index for small-cell carcinoma was significantly higher than that for adenocarcinoma ($P = 0.0230$). In addition, when the cut-off value was set at 7.2, which corresponded to twice the 95% specificity level for normal volunteers, the sensitivities were 46.1% for all lung cancer, 28.0% for adenocarcinoma, 55.6% for squamous cell carcinoma, 100% for large-cell carcinoma and 71.4% for small-cell carcinoma (Figure 1). With this cut-off value, the sensitivity of the anti-p53 index for squamous cell carcinoma was significantly higher than that for adenocarcinoma ($P = 0.0359$).

The ROC curve for the anti-p53 index for patients with lung cancer is shown in Figure 3. This curve demonstrated that the serum anti-p53 index had both high sensitivity and specificity for the detection of lung cancer in this study. This index thus appears to be useful as a marker of lung cancer at its initial presentation.

### Multivariate analysis of factors associated with elevated anti-p53 index level in sera of lung cancer patients

The factors associated with elevated anti-p53 index level (≥ 3.6, determined using the 95% specificity level for normal volunteers in this study) for lung cancer patients were further assessed using logistic regression analysis. All the parameters listed in Table 1 were included and analysed in backward step-wise fashion. The model finally selected [$\chi^2$ (5) = 19.6, $P = 0.0015$] is shown in Table 2. In this model, histology was a dominant factor associated with elevated anti-p53 index level ($P = 0.0255$). Gender, age and smoking index were selected as factors influencing index level, but their probabilities did not reach significance ($P = 0.0586$, $P = 0.0847$, $P = 0.0911$ respectively). In addition, previous or concomitant cancer was selected as a reverse factor. Disease stage was not selected in this regression model. Furthermore, in the model using the cut-off value of 7.2 [$\chi^2$ (1) = 6.9, $P = 0.0084$], histology was a dominant factor associated with elevated anti-p53 index level ($P = 0.0184$) (Table 2).

### DISCUSSION

In our prospective study using a series of serum samples obtained from patients newly diagnosed with lung cancer and normal

© Cancer Research Campaign 1998

Table 2 Multivariate analysis of factors associated with elevated serum anti-p53 antibody index level in lung cancer patients

| Parameters | β | s.e. | Wald | P-value |
|---|---|---|---|---|
| Cut-off level ≥ 3.6 | | | | |
| Histology (adeno vs squamous vs large vs small) | 1.2717 | 0.5692 | 4.9919 | 0.0255 |
| Gender (male vs female) | 3.7045 | 1.9591 | 3.5756 | 0.0586 |
| Presence of previous or concomitant cancer (no vs yes) | −2.0188 | 1.1499 | 3.0822 | 0.0792 |
| Age (< 68 years vs ≥ 68) | 1.3240 | 0.7681 | 2.9714 | 0.0847 |
| Smoking index (< 600 vs ≥ 600) | 3.0217 | 1.7885 | 2.8544 | 0.0911 |
| Cut-off level ≥ 7.2 | | | | |
| Histology (adeno vs squamous vs large vs small) | 0.8013 | 0.3398 | 5.5594 | 0.0184 |

Elevated serum anti-p53 index level was defined as ≥ 3.6 based on the 95% specificity level for normal volunteers. Analysis using the cut-off level of 7.2, which corresponded to twice the 95% specificity level, was also performed. The categorized variables for each parameter were encoded as 0, 1, 2 and 3 in the left order in this logistic regression analysis. Abbreviations: adeno, adenocarcinoma; large, large-cell carcinoma; small, small-cell carcinoma; squamous, squamous cell carcinoma.

volunteers, serum anti-p53 level was found to be higher in lung cancer patients than in normal volunteers. For lung cancer patients, a significant difference in index levels was found by histological type of tumour: based on the cut-off value corresponding to twice the 95% specificity level for normal volunteers, elevated anti-p53 levels were found in 28.0% of patients with adenocarcinoma, 55.6% of those with squamous cell carcinoma, 100% of those with large-cell carcinoma and 71.4% of those with small-cell carcinoma. These incidences were higher than those noted in previous studies including various types of cancer (Crawfort et al, 1982; Caron de Fromentel et al, 1987; Davidoff et al, 1992; Schlichtholz et al, 1992, 1994; Winter et al, 1992, 1993; Labrecque et al, 1993; Lubin et al, 1993; Green et al, 1994; Mudenda et al, 1994; Muller et al, 1994; Chang et al, 1995). In a lung cancer series, Schlichtholz et al (1994) reported that anti-p53 antibodies were found in sera in 20.0% of patients with adenocarcinoma, 11.1% of those with squamous cell carcinoma, 40.0% of those with large-cell carcinoma and 44.4% of those with small-cell carcinoma. This difference in incidences from those in our study is clearly due to differences in the criteria used in the two studies. Schlichtholz et al (1994) defined a 'positive' finding of anti-p53 in serum as absorbance at least equal to that of anti-p53-positive control serum, which was demonstrated to have anti-p53 by both immunoprecipitation and immunoblotting assays. However, in our study, 'elevated' anti-p53 level was defined in accordance with the 95% specificity approach for normal volunteers. The criteria used by Schlichtholz et al (1994) are valid. In fact, presence of anti-p53 was confirmed only for sera with a high anti-p53 index level in our immunoblotting assay. However, the frequency of false-negative findings for anti-p53 might be increased with the use of these criteria, as, in general, immunoblotting and immunoprecipitation assays have lower sensitivity for detection of target molecules than does ELISA. In addition, according to two independent studies (Schlichtholz et al, 1994; Trivers et al, 1996) as well as our own, anti-p53 levels determined by ELISA are continuously plotted for cancer patients and appear to be higher than those in normal control subjects. We therefore believe that the 95% specificity approach may be clinically useful for the detection of anti-p53.

Concerning the relationship between elevated anti-p53 levels and clinical features of lung cancer patients, histological type of tumour was found to be a dominant factor affecting elevation of anti-p53 level in our logistic regression model. Large-cell carcinoma and small-cell carcinoma both demonstrated elevated anti-p53 levels. This finding is similar to that reported by Schlichtholz et al (1994), and is supported by the finding of high *p53* gene mutation frequencies in lung cancer tumours, e.g. 55% for non-small-cell lung cancer tumours and 78% for small-cell lung cancer tumours (Johnson, 1995). These findings indicate that serum anti-p53 index level is a marker for both large-cell carcinoma and small-cell carcinoma, and, that is to say, its elevation might be correlated with tumour histology showing rapid growth. However, no relationship was found between elevated serum anti-p53 index level and disease stage. We therefore speculate that anti-p53 index level might be independent of tumour burden and disease stage in cancer patients, based on the following findings: (1) that emergence of serum anti-p53 was demonstrated before clinical detection of cancers in a subset of a non-cancer cohort (Lubin et al, 1995; Trivers et al, 1995, 1996); (2) that serum levels of anti-p53 remained constant in cancer patients if curative treatment could not be performed (Schlichtholz et al, 1994). Notably, the anti-p53 serum index has the potential for use in early diagnosis of cancers. The presence of *p53* gene mutation has been demonstrated in premalignant bronchial lesions such as mild and severe epithelial dysplasias (Sundaresan et al, 1992). Therefore, heavy smoking, which leads to bronchial epithelial dysplasia, might elevate serum anti-p53 index level. In addition, in our study, no correlation was found between elevated serum anti-p53 level and the presence of previous or concomitant cancer, although Mudenda et al (1994) did find a positive correlation between these two. A large cohort study will be needed to determine the true nature of this correlation.

In conclusion, in our study using a series of serum samples from patients newly diagnosed with lung cancer, serum anti-p53 index level appeared possibly to be independent of tumour burden and the presence of previous or concomitant cancer, but was strongly correlated with tumour histological type.

### REFERENCES

Caron de Fromental C, May-Levin F, Mouriesse H, Lemerle J, Chandrasekaran K and May P (1987) Presence of circulating antibodies against cellular protein p53 in a notable proportion of children with B-cell lymphoma. *Int J Cancer* **39**: 185–189

Chang F, Syrjänen S and Syrjänen K (1995) Implications of the p53 tumour-suppressor gene in clinical oncology. *J Clin Oncol* **13**: 1009–1022

Crawfort LV, Pim DC and Bulbrook RD (1982) Detection of antibodies against the cellular protein p53 in sera from patients with breast cancer. *Int J Cancer* **30**: 403–408

Davidoff AM, Iglehart JD and Marks JR (1992) Immune response to p53 is dependent upon p53/HSP70 complexes in breast cancers. *Proc Natl Acad Sci USA* **89**: 3439–3442

Green JA. Mudenda B. Jenkins J. Leinster SJ. Tarunina M. Green B and Robertson L (1994) Serum p53 autoantibodies: incidence in familial breast cancer. *Eur J Cancer* **30A**: 580–584

Harris CC and Hollstein M (1993) Clinical implications of the p53 tumor-suppressor gene. *N Engl J Med* **329**: 1318–1327

Hollstein M. Sidransky D. Vogelstein B and Hallis CC (1991) p53 mutations in human cancers. *Science* **253**: 49–53

Johnson BE (1995) Biology of lung cancer. In *Lung Cancer*. Johnson BE and Johnson DH (eds). pp. 15–40. Wiley-Liss: New York

Klapdor R (1992) Arbeitsgruppe Qualitätskontrolle und Standardisierung von Tumormarkertests im Rahmen der Hamburger Symposien über Tumormarker (in German). *Tumordigan u Ther* **13**: 19–22

Labrecque S. Naor N. Thomson D and Matlashewski G (1993) Analysis of the anti-p53 antibody response in cancer patients. *Cancer Res* **53**: 3468–3471

Levine AJ. Momand J and Finlay CA (1991) The p53 tumour suppressor gene. *Nature* **351**: 453–456

Lubin R. Schlichtholz B. Bengoufa D. Zalcman G. Trédaniel J. Hirsch A. Caron de Fromental C. Preudhomme C. Fenaux P. Fournier G. Mangin P. Laurent-Puig P. Pelletier G. Schlumberger M. Desgrandchamps F. Le Duc A. Peyrat JP. Janin N. Bressac B and Soussi T (1993) Analysis of p53 antibodies in patients with various cancers define B-cell epitopes of human p53: distribution of primary structure and exposure on protein surface. *Cancer Res* **53**: 5872–5876

Lubin R. Zalcman G. Bouchet L. Trédaniel J. Legros Y. Cazals D. Hirsch A and Soussi T (1995) Serum p53 antibodies as early markers of lung cancer. *Nature Med* **1**: 701–702

Metz CE (1991) *CLABROC User's Guide: Apple Macintosh™ Version*. The University of Chicago: Chicago

Mountain CF (1986) A new international staging system for lung cancer. *Chest* **89**: 225S–233S

Mudenda B. Green JA. Green B. Jenkins JR. Robertson L. Tarunina M and Leinster SJ (1994) The relationship between serum p53 autoantibodies and characteristics of human breast cancer. *Br J Cancer* **69**: 1115–1119

Muller M. Volkmann M. Zentgraf H and Galle PR (1994) Clinical implications of the p53 tumor-suppressor gene. *N Engl J Med* **330**: 865

Peyrat J. Bonneterre J. Lubin R. Vanlemmens L. Fournier J and Soussi T (1995) Prognostic significance of circulating p53 antibodies in patients undergoing surgery for locoregional breast cancer. *Lancet* **345**: 621–622

Schlichtholz B. Legros Y. Gillet D. Gaillard C. Marty M. Lane D. Calvo F and Soussi T (1992) The immune response to p53 in breast cancer patients is directed against immunodominant epitopes unrelated to the mutational hot spot. *Cancer Res* **52**: 6380–6384

Schlichtholz B. Trédaniel J. Lubin R. Zalcman G. Hirsch A and Soussi T (1994) Analyses of p53 antibodies in sera of patients with lung carcinoma define immunodominant regions in the p53 protein. *Br J Cancer* **69**: 809–816

Segawa Y. Takigawa N. Mandai K. Maeda Y. Takata I. Fujimoto N and Jinno K (1997*a*) Measurement of serum p53 protein in patients with small cell lung cancer and results of its clinicopathological evaluation. *Lung Cancer* **16**: 229–238

Segawa Y. Takigawa N. Kataoka M. Takata I. Fujimoto N and Ueoka H (1997*b*) Risk factors for development of radiation pneumonitis following radiation therapy with or without chemotherapy for lung cancer. *Int J Radiat Oncol Biol Phys* **39**: 91–98

Sundaresan V. Ganly P. Hasleton P. Rudd R. Sinha G. Bleehen NM and Rabbitts P (1992) p53 and chromosome 3 abnormalities, characteristic of malignant lung tumours, are detectable in preinvasive lesions of the bronchus. *Oncogene* **7**: 1989–1997

Trivers GE. Cawley HL. De Benedetti VM. Hollstein M. Marion MJ. Bennett WP. Hoover ML. Prives CC. Tamburro CC and Harris CC (1995) Anti-p53 antibodies in sera of workers occupationally exposed to vinyl chloride. *J Natl Cancer Inst* **87**: 1400–1407

Trivers GE. De Benedetti VM. Cawley HL. Caron G. Harrington AM. Bennett WP. Jett JR. Colby TV. Tazelaar H. Pairolero P. Miller RD and Harris CC (1996) Anti-p53 antibodies in sera from patients with chronic obstructive pulmonary disease can predate a diagnosis of cancer. *Clin Cancer Res* **2**: 1767–1775

Winter SF. Minna JD. Johnson BE. Takahashi T. Gazdar AF and Carbone DP (1992) Development of antibodies against p53 in lung cancer patients appears to be dependent on the type of p53 mutation. *Cancer Res* **52**: 4168–4174

Winter SF. Sekido Y. Minna JD. McIntire D. Johnson BE. Gazdar AF and Carbone DP (1993) Antibodies against autologous tumor cell proteins in patients with small-cell lung cancer: association with improved survival. *J Natl Cancer Inst* **85**: 2012–2018

World Health Organization (1982) Histological typing of lung tumours. 2nd edn. *Am J Clin Pathol* **77**: 123–136

© Cancer Research Campaign 1998